# Non-invasive paper-based microfluidic device for ultra-low detection of urea through enzyme catalysis

**DOI:** 10.1098/rsos.171980

**Published:** 2018-03-21

**Authors:** Vignesh Suresh, Ong Qunya, Bera Lakshmi Kanta, Lee Yeong Yuh, Karen S. L. Chong

**Affiliations:** Institute of Materials Research and Engineering (IMRE), Agency for Science, Technology and Research (A*STAR), #08-03, 2 Fusionopolis Way, Innovis, Singapore 138634, Republic of Singapore

**Keywords:** paper fluidics, ultra-low detection, urea, point-of-care, chemical sensor

## Abstract

This work describes the design, fabrication and characterization of a paper-based microfluidic device for ultra-low detection of urea through enzyme catalysis. The microfluidic system comprises an entry port, a fluidic channel, a reaction zone and two electrodes (contacts). Wax printing was used to create fluidic channels on the surface of a chromatography paper. Pre-conceptualized designs of the fluidic channel are wax-printed on the paper substrate while the electrodes are screen-printed. The paper printed with wax is heated to cause the wax reflow along the thickness of the paper that selectively creates hydrophilic and hydrophobic zones inside the paper. Urease immobilized in the reaction zone catalyses urea into releasing ions and, thereby, generating a current flow between the electrodes. A measure of current with respect to time at a fixed potential enables the detection of urea. The methodology enabled urea concentration down to 1 pM to be detected. The significance of this work lies in the use of simple and inexpensive paper-based substrates to achieve detection of ultra-low concentrations of analytes such as urea. The process is non-invasive and employs a less cumbersome two-electrode assembly.

## 1. Introduction

Recent advances in microfluidics present different material substrates such as polymers, paper and overhead transparency sheets that enable controlled flow of fluid through micro-channels and, thus, are not limited to device channels conventionally made using polydimethylsiloxane (PDMS) [1–3]. In the last few years, there has been a tremendous growth in fluid flow dynamics, process optimization, analyte detection, device fabrication and electronics integration for microfluidics that have enabled the use of microfluidics in healthcare, forensic analysis, medical diagnostics and environmental monitoring applications [4–9]. Microfluidics integrated with other prominent sensing techniques such as surface-enhanced Raman scattering enable label-free biomolecular detection with unprecedented detection limit [10].

Among the materials to fabricate microfluidic substrates, plastics and papers are the most promising choices for point-of-care devices that need to be in contact with bodily fluids such as urine, serum and blood owing to their biocompatibility, flexibility and ease of handling [11–13]. In particular, microfluidics made using paper have gained much interest, as fluid flow by capillary action can be engineered through the fabrication of hydrophilic paper channels bordered by hydrophobic wax/resist barriers. Paper-based microfluidic devices have emerged as a new class of point-of-care diagnostic devices that are biocompatible, inexpensive, flexible, easy to fabricate and dispose [14–18].

Patterning of micro-channels via wax printing can be easily accomplished in a non-clean-room environment using a wax printer, unlike conventional, complex silicon-based micro-fabrication processes, such as photolithography, that are expensive. Furthermore, wax printing allows complex three-dimensional (3D) microfluidic configurations. These can be achieved either by stacking multiple layers of wax-patterned paper substrates, where a channel is established when the hydrophilic regions in different layers are interconnected, or by controlling the penetration depth of melted wax on both sides of the same paper substrate to form 3D hydrophobic barriers [19–21].

There are reports on the fabrication of paper-based micro-channels using photolithography whereby photoresists serve as barriers on paper substrates [15,22]. Inkjet printing, flexographic printing, UV–ozone etching and plasma etching techniques have also been used to pattern paper substrates to create hydrophilic channels [17,23–27]. Although these techniques have been effective in generating micro-scale geometries, they are time-consuming and complex, requiring specialized infrastructure such as a clean-room environment. On the other hand, creating micro-channels in paper through wax printing is relatively simple and inexpensive [28,29]. Dungchai *et al.* [30] reported the first demonstration of electrochemical detection for paper-based microfluidic devices. Since then, the use of paper-based micro-channels in analytical chemistry has spread far and wide. The state-of-the-art in paper fluidics has been the translation of nucleic acid analysis to a paper-based format, smartphone-based analysis, male fertility testing and wearable diagnostics [18,31,32].

Recently, paper-based microfluidics have been explored to detect analytes such as urea and glucose with high precision at low cost [16,17,19,33]. Urea sensors find applications that extend all the way from healthcare and diagnostics, to phytoplankton physiology and ecology [34,35]. Processed water generated from urea plants and the accidental seepage of urea from agricultural lands into the water streams pose major threats to ecology causing water pollution [36]. Thus, ultra-low detection of urea is particularly important for environmental monitoring, water filtration and quality control.

Most of the existing urea sensors employ electrochemical techniques with a three-electrode system, viz. a working electrode, a counter electrode and a reference electrode. A urea biochemical sensor capable of detecting 0.316 mM with a sensitivity of 31.12 mV/log[*M*] has been recently reported [37]. However, in the present work, paper-based microfluidic devices are fabricated to detect ultra-low concentrations of urea using a two-electrode assembly. Urease, immobilized in the reaction zone, catalyses the breakdown of urea to ions that give rise to current. Although colorimetric assays and the electrochemical measurements may be suitable for several analytical applications, a simple measure of current generated by the ions in aqueous product after enzyme catalysis using a two-electrode system offers ultra-low detection of urea samples [38]. On a similar note, capturing enzymatic reactions at nanochannels using simple conductance measurements that reflect the movement of ions between two electrodes has also been reported [39]. Moreover, a simple two-electrode configuration also eliminates the problems associated with the need for constant surface area of the counter electrode for accurate conductivity measurement in a three-electrode electrochemical system. The resulting paper-based microfluidics device offers ease in handling and exhibits excellent reproducibility. It can further be extended to a range of other analytes that undergo enzyme catalysis.

## 2. Experimental

### 2.1. Materials

Filter paper (No. 50, 0.25 mm thick, Advantec, Japan) was used in device fabrication. Urease from *Canavalia ensiformis* (U4002, Sigma Aldrich) was prepared in phosphate-buffered saline to a concentration of 50 mg ml^−1^. Urea stock solution was prepared by dissolving urea powder (1st BASE, Singapore) in ultrapure water to a working concentration of 1 M. Urea solutions with various lower concentrations were prepared by further dilution of the stock solution with ultrapure water. As urease was unstable at room temperature, the hydrolysis of urea was carried out as soon as the enzymes were immobilized. Test pads on the reagents strips for urinalysis (Multistix^®^ 8 SG, Siemens Healthcare Diagnostics, Australia) were used to prepare the device that allowed colorimetric detection of multiple analytes. A test solution of multiple analytes comprising 50 g l^−1^ d-glucose (1st BASE, Singapore) and 1 g l^−1^ albumin (human, F-V, lyophilized, Nacalai Tesque, Japan) was prepared in deionized water.

### 2.2. Methods

#### 2.2.1. Fabrication of paper-fluidic device

**Figure 1.**
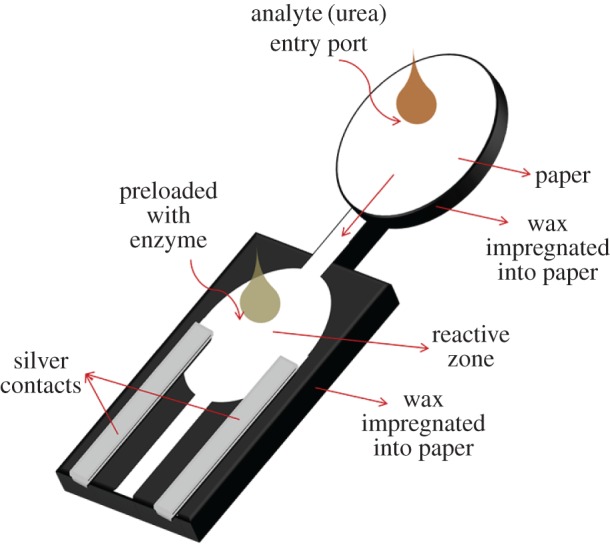
Schematic illustration of the paper-based microfluidic device that enables ultra-low detection of analytes (urea). Analytes enter through the entry port, and flow through the channel via capillary action, before reaching the reaction zone, which is preloaded with enzymes (urease). An enzymatic reaction takes place in the reaction zone, generating ions that result in current flow between the electrodes. The electrodes are screen printed using silver paste.

Doubled-sided printing on the filter paper (whatman equivalent 20Chr) was performed using a wax printer (ColorQube 8570, Xerox). Channel designs were printed on the top surface as shown in the schematic illustration (figure 1), while the bottom of the paper was fully printed in wax to render it hydrophobic. This not only prevents leakage of the analyte from the bottom, but also reduces the sample volume that flows through the capillary. The wax-printed paper substrate was heated on a hot plate at 85°C for 2 min. Wax on both sides of the paper then began to flow and penetrate into the paper. The temperature and timing were critical to determine the depth of penetration of the wax into the paper. The conditions were optimized such that the wax on both sides only penetrated halfway through the paper. Areas with wax printed on both sides would have complete impregnation of wax along the width of the paper. The wax-filled regions become hydrophobic, while the regions without wax (paper) remain hydrophilic and form the fluidic channels lined by wax.

**Figure 2.**
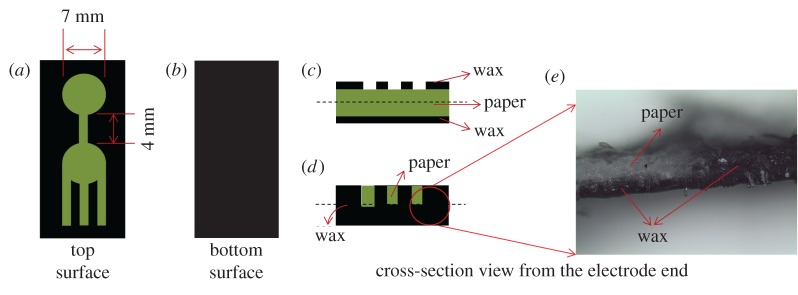
(*a*) A schematic illustrating the top surface of the wax-printed paper. The reaction zone and the entry port have diameters of 7 mm, while the interconnecting channel measures 4 mm in length and 1.5 mm in width. (*b*) The bottom surface of the paper is entirely wax printed to render it hydrophobic and to prevent leakage. (*c*,*d*) Cross-sections of the device from the electrode-end before and after heating, respectively. Note that the wax on both sides has melted and impregnated into the paper as evidenced by the photograph in (*e*). Paper regions are coloured green for easy visualization.

For applications that need potential at each of the electrodes to be measured as in a three-electrode electrochemical system, a third electrode can also be integrated onto the device. However, the proposed simple two-electrode set-up will be adequate to detect ultra-low concentration of analytes as it is able to capture even minuscule movement of ions responsible for the closed-circuit current. In this work, silver paste was screen printed onto the hydrophilic region of the paper substrate to serve as the metallic contact in the two-electrode configuration (figure 1). The geometrical dimensions of the wax-printed paper are shown in figure 2. The distance between the two electrodes is 4 mm. The reaction zone and the entry port have diameters of 7 mm.

To fabricate the device for detection of urea, urease was immobilized in the reaction zone by preloading 7 mm diameter paper discs with 10 µl of 50 mg ml^−1^ of urease. The enzyme-loaded discs were dried before placing them on the reaction zone together with a smaller disc of pH indicator cut out from pH indicator strips. The assembly was then secured in place with an inert tape. The reaction zone was connected to the analyte entry port through a small hydrophilic channel 1.5 mm in width and 4 mm in length.

#### 2.2.2. Detection of urea

**Figure 3.**
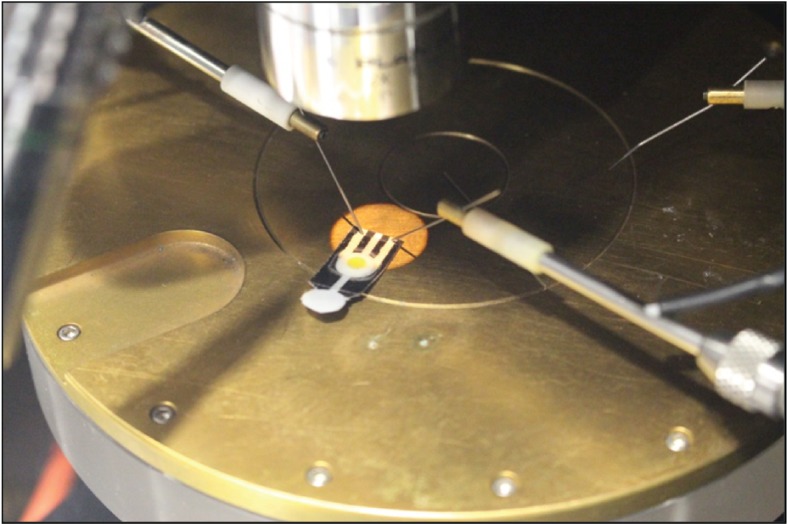
Current profile was measured using a semiconductor parameter analyser. Paper-based microfluidic devices immobilized with urease were tested with samples containing 1 mM, 1 µM, 1 nm and 1 pM of urea.

Thirty microlitre aqueous samples of different urea concentrations, 1 mM, 1 µM, 1 nM and 1 pM, were injected into the entry port and were allowed to reach the reaction zone by capillary action where enzyme catalysis hydrolysed urea. For each sample, change in pH as shown by the pH indicator was noted and the current profile between the two electrodes was recorded using a semiconductor parameter analyser (Agilent 4156C). The semiconductor parameter analyser was set to the following conditions prior to measurement—electrode bias 2 V, current compliance 40 mA, time 20 min. The two point probes were gently pressed onto the silver electrodes. The measurement was started without introduction of any analyte sample (figure 3). After 100 s, 30 µl of sample was immediately injected into the entry port and allowed to reach the enzyme-loaded reaction zone by capillary action. The change in current with respect to time was measured. A control run was carried out using a substrate that was not loaded with urease. Through the entry port, 30 µl of 1 mM urea was dispensed and the above-mentioned measurements were carried out.

#### 2.2.3. Colorimetric detection of multiple analytes

The device for colorimetric detection of multiple analytes was fabricated in a similar manner as the device for detection of urea. Instead of using urease-loaded discs, 6 mm discs were punched out from the test pads on the reagents strips for urinalysis, and placed on the reaction zones of the wax-printed paper. Fifty microlitres of a test solution containing 50 g l^−1^ glucose and 1 g l^−1^ albumin was injected into the entry port and allowed to reach the reaction zones by capillary action. Colorimetric changes in the reaction zones were inspected visually.

## 3. Results and discussion

A paper-based device comprising an entry port, a fluidic channel, a reaction zone and two electrodes was fabricated (figure 1). Samples containing urea were injected into the entry port and the fluid flowed through the channel via capillary action, before reaching the reaction zone, which was preloaded with urease. In the course of enzymatic hydrolysis of urea, ammonium carbonate was produced, which dissociated into several ions, and the reaction could be monitored by measuring the current flow between the electrodes [37].

$${\text{H}}_{2}\text{N}-\mathrm{CO}-\text{N}{\text{H}}_{2}+3{\text{H}}_{2}\text{O }+{\text{H}}^{+}\overset{\mathrm{urease}}{\to }2\;\text{N}{{\text{H}}_{4}}^{+}+\text{O}{\text{H}}^{-}+\text{HC}{{\text{O}}_{3}}^{-}.$$

**Figure 4.**
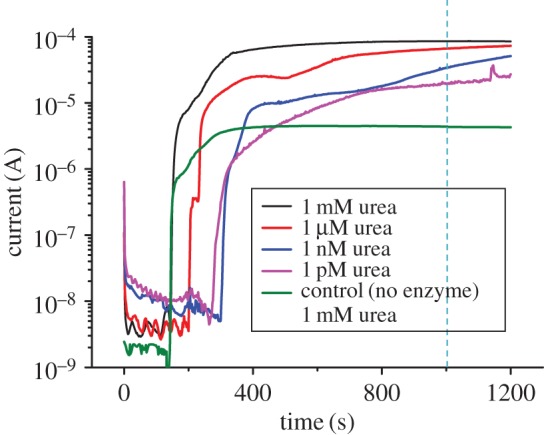
Current–time profile of samples with different urea concentrations. The control sample had no pre-loaded enzyme and was tested using 30 µl of 1 mM urea as analyte.

In figure 4, the current profiles with respect to time from single devices at each concentration are presented. It could be observed from the current–time plot that there was a sudden sharp increase in current when the electrodes came in contact with the liquid front. Urea in aqueous solution is generally electrolytically conducting [40]. As the liquid front reached the reaction zone, urea was catalysed by urease into releasing ammonium ions. Ions in the liquid closed the circuit between the electrodes and the current increased as the enzymatic reaction took place. As long as the fluid transported by capillary action continued to flow through the hydrophilic channels, the contact established with the electrodes captured the minuscule change in conductivity. The measured currents, therefore, do not arise from bulk fluid flow as observed in traditional microfluidic channel where fluid was flown in bulk through the conduit. Furthermore, wax is an electrical insulator and offers excellent barrier to polar analytes. It also prevents the analyte from leaking onto the metal plate. Therefore, the values reported are not the current passing through the wet paper via the bottom metal plate. Eventually, the current plateaued over time and remained stable as long as the paper remained wet. Evaporation was mitigated by carefully covering the substrate with inert tape. With regard to the time variation in the onset of current, we believe it is due to the inherent porous nature of the paper substrate in being non-uniform and non-homogeneous. Therefore, the onset current is slightly different for different devices.

**Figure 5.**
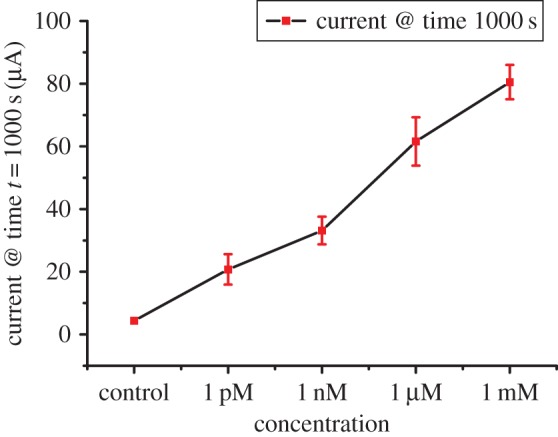
Plot of average current measured at *t* = 1000 s between three devices versus the urea concentration of the samples. The current measured increased linearly with analyte concentration. Devices tested for analyte concentration of 1 mM, 1 µM, 1 nM and 1 pM showed a maximum variation of 5.5%, 7.7%, 4.4% and 4.8%, respectively.

At the lowest urea concentration of 1 pM, the current measured was approximately 19 µA; that is significantly higher and can be differentiated from the control. However, detection for concentrations less than 1 pM became increasingly difficult as the current measured was similar to control and cannot be resolved. Furthermore, the results were not consistent, probably due to the concerns over preparing an accurate concentration of analytes at such lower concentration levels. However, the limit of detection and range of urea sensors can be customized based on the requirements. Repeatability tests were performed to ensure the reproducibility of the results. The performance of the devices for different analyte concentrations is reported in electronic supplementary material, figure S1. Currents measured at 1000 s were averaged for all devices and plotted against their corresponding urea concentrations (figure 5). The devices with varying urea concentrations showed standard deviation below 10%.

While sensors for measuring urea in urine, blood and serum need to be able to pick up concentrations ranging from a few micromolar to several millimolar, sensors for trace urea detection need to have ultra-low detection limit ranging from a few nanomolar to picomolar [34,41–43]. Thus, the technology as a whole can be extended to various grades of paper and other analytes contributing to enzyme kinetics.

**Figure 6.**
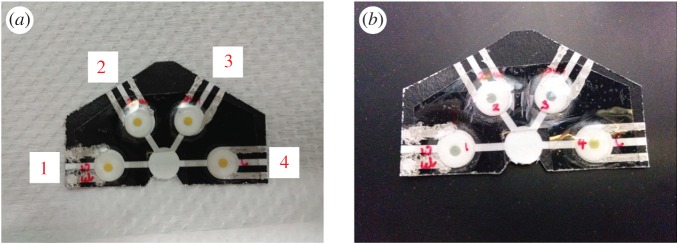
Photos of a multi-channel fluidic system fabricated for simultaneous detection of analytes. The as-prepared substrates with pre-loaded urease of concentration 50 mg ml^−1^ and pH marker are seen in (*a*). From left to right (1–4), the volume of urease immobilized on the reaction zone was 10, 20, 20 and 0 µl (control). (*b*) The multi-device after the test was performed using 30 µl of 1 M urea dispensed in the common port of entry. From left to right (1–4), the first three reaction zones shows a change in colour indicating that enzymatic reaction had taken place. The fourth reaction zone shows no colour change expected from a substrate with no pre-loaded enzyme.

The paper-based microfluidics system can be improvised for simultaneous detection of analytes by adding multiple reaction zones that share a common entry port. Response of the injected analyte under different reaction conditions can be studied by varying parameters such as enzyme concentration, injection volume and pH. Figure 6 shows one such multi-channel fluidic system fabricated to demonstrate the versatility of our system. Reaction zones in the device in figure 6*a* were immobilized with urease of varying volumes 10, 20, 20 and 0 µl (control), from left to right. A pH marker was attached to each reaction zone. The pH indicator discs initially appeared yellow before sample injection. All, except the control with no urease, changed colour (figure 6*b*) after 30 µl of 1 M urea was introduced. This suggested that the release of ions was due to the catalysis of urea by urease. The control reaction zone had no enzyme, thus no enzymatic reaction occurred and pH remained unchanged. More information on the colorimetry study and the advantages of the paper-based device can be found in electronic supplementary material, figure S2.

**Figure 7.**
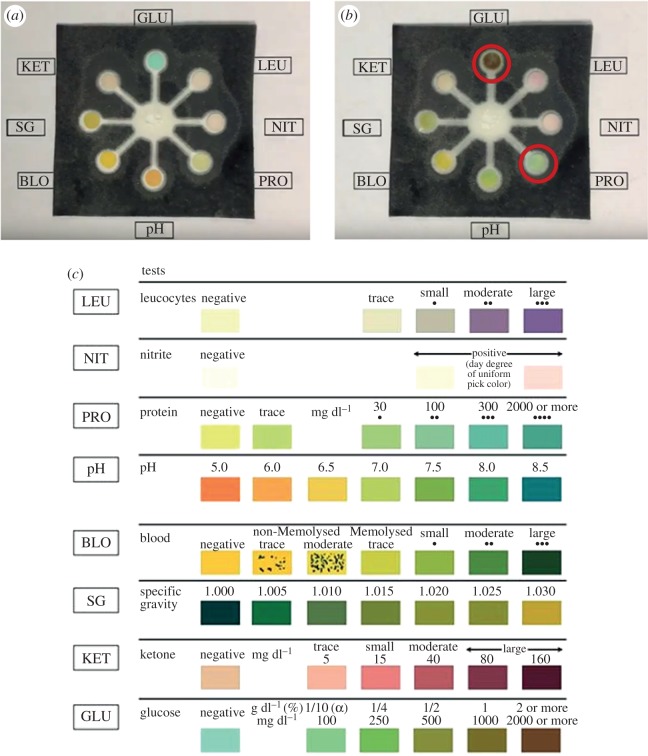
A paper-based point-of-care testing device that enables simultaneous colorimetric detection of multiple analytes. (*a*) The paper-based device can be designed to accommodate eight reaction zones for detection of eight different analytes and properties—leucocytes (LEU), nitrite (NIT), protein (PRO), pH, blood (BLO), specific gravity (SG), ketone (KET) and glucose (GLU). (*b*) Changes in colour were observed in the reaction zones for detection of glucose and protein (circled in red) after the addition of a test solution containing glucose and albumin. (*c*) Colour changes at the reaction zones can be compared to the colour chart from the manufacturer of the reagents strips for urinalysis, to determine the quantities of the various analytes present.

We have demonstrated the capability of our paper-fluidic technology in measuring several analytes simultaneously by fabricating a multi-channel device for colorimetric detection of multiple analytes (figure 7*a*). This type of device can be used in point-of-care testing. Test pads, impregnated with the relevant detection reagents for the various analytes, were cut out from commercial reagents strips for urinalysis. The test pad for detection of protein contains tetrabromophenol blue, an acid–base indicator. Protein is detected based on the protein-error-of-indicators principle—the ability of protein to change the colour of some acid–base indicators without altering the pH. The test pad for detection of glucose contains glucose oxidase, horseradish peroxidase and potassium iodide. Glucose oxidase catalyses the formation of gluconic acid and hydrogen peroxide from the oxidation of glucose. This is followed by a reaction catalysed by horseradish peroxidase, whereby the hydrogen peroxide produced oxidizes potassium iodide, a chromogen, to result in a colour change. When 50 µl of a test solution containing 50 g l^−1^ glucose and 1 g l^−1^ albumin was injected into the entry port, colour changes were observed in the reaction zones for detection of glucose and protein. The disc for glucose detection changed from green to brown while that for protein detection altered from yellow to light green (figure 7*b*). The colour chart from the manufacturer of the reagents strips for urinalysis shows the expected changes in colour corresponding to the varying amounts of different analytes (figure 7*c*). This suggests that changes in colour can be calibrated to give a rough quantitative indication of the analyte present (figure 7*c*). In our experiment, the colour changes observed were compared with the manufacturer's colour chart and found to correspond well with the actual amount of glucose and albumin present in the test solution. Therefore, we have shown that multiple analytes in a small-volume sample can be detected simultaneously and quantitatively to a certain extent, using our paper-fluidic technology. The addition of electrodes to this device can potentially improve the sensitivity and accuracy of measurements.

## 4. Conclusion

We have demonstrated the fabrication of a low-cost paper-based microfluidic device using wax printing is simple and versatile. It eliminates the need for complex processes associated with traditional PDMS-based microfluidic devices. As paper channels allow fluid flow by capillary action rather than free flow through conduits associated with PDMS channels, they avoid the problem of air traps which can introduce inaccuracies in analyte detection. The paper-based microfluidic device makes use of the laboratory-scale parameter analyser to measure current that arises from ions generated during enzyme catalysis. It represents a non-invasive electronic sensing system for chemical detection using a very cost-effective fabrication strategy by using paper substrates and wax printing. On those occasions when colorimetric detection induce uncertainty, the change in the ion concentrations still remains highly sensitive and, thereby, enables ultra-low level detection of analytes at picomolar scale or less. Furthermore, this device can be used to measure other analytes such as glucose, sodium lactate etc.

Our method of fabrication can also be easily extended to build a multi-fluidic channel system that allows simultaneous detection of ions released during different reaction conditions. Paper-based substrate has the advantage of being disposable and environmentally friendly. The low cost of production involved in making this device will also encourage the adoption of this technology in resource-scarce settings.

## Supplementary Material

Repeatability tests were performed to ensure the reproducibility of the results. The performance of the devices for different urea concentrations (1mM, 1µM, 1nm and 1pM) is reported in the supporting information

## Supplementary Material

The analyte after being dispensed (A) flows through the paper channels and react with the test reagents in the ports (B) resulting in color change (C). The paper based devices are ultra-thin (D) and are highly flexible (E) that they are often the preferred candidates in point-of-care applications.

## Data accessibility

Device reproducibility test (electronic supplementary material, figure S1) and fluid transport through the multi-channel paper fluidic device (electronic supplementary material, figure S2). The datasets supporting this article have been uploaded as part of the electronic supplementary material.
