# Supplementary material for: Non-invasive paper-based microfluidic device for ultra-low detection of urea through enzyme catalysis
Source: R Soc Open Sci. 2018 Mar 21;5(3):171980. doi: 10.1098/rsos.171980 (PMC5882721; doi:10.1098/rsos.171980)
Supplement: Repeatability tests were performed to ensure the reproducibility of the results. The performance of the devices for different urea concentrations (1mM, 1µM, 1nm and 1pM) is reported in the supporting information [file rsos171980supp1.docx]

**Supporting Information**

Non-Invasive Paper-Based Microfluidic Device for Ultra-Low Detection of Urea through Enzyme Catalysis

Vignesh Suresh, Ong Qunya, Bera Lakshmi Kanta, Lee Yeong Yuh and Karen S.L. Chong*

*[karen-chong@imre.a-star.edu.sg](mailto:karen-chong@imre.a-star.edu.sg)

**Figure S1.** Plots of current vs time performed to demonstrate the repeatability and reproducibility of the results of the fluidic devices in sensing urea.
