# Supplementary material for: Non-invasive paper-based microfluidic device for ultra-low detection of urea through enzyme catalysis
Source: R Soc Open Sci. 2018 Mar 21;5(3):171980. doi: 10.1098/rsos.171980 (PMC5882721; doi:10.1098/rsos.171980)
Supplement: The analyte after being dispensed (A) flows through the paper channels and react with the test reagents in the ports (B) resulting in color change (C). The paper based devices are ultra-thin (D) and are highly flexible (E) that they are often the preferred candidates in point-of-care applications. [file rsos171980supp2.docx]

**Supporting Information**

Non-Invasive Paper-Based Microfluidic Device for Ultra-Low Detection of Urea through Enzyme Catalysis

Vignesh Suresh, Ong Qunya, Bera Lakshmi Kanta, Lee Yeong Yuh and Karen S.L. Chong*

*[karen-chong@imre.a-star.edu.sg](mailto:karen-chong@imre.a-star.edu.sg)


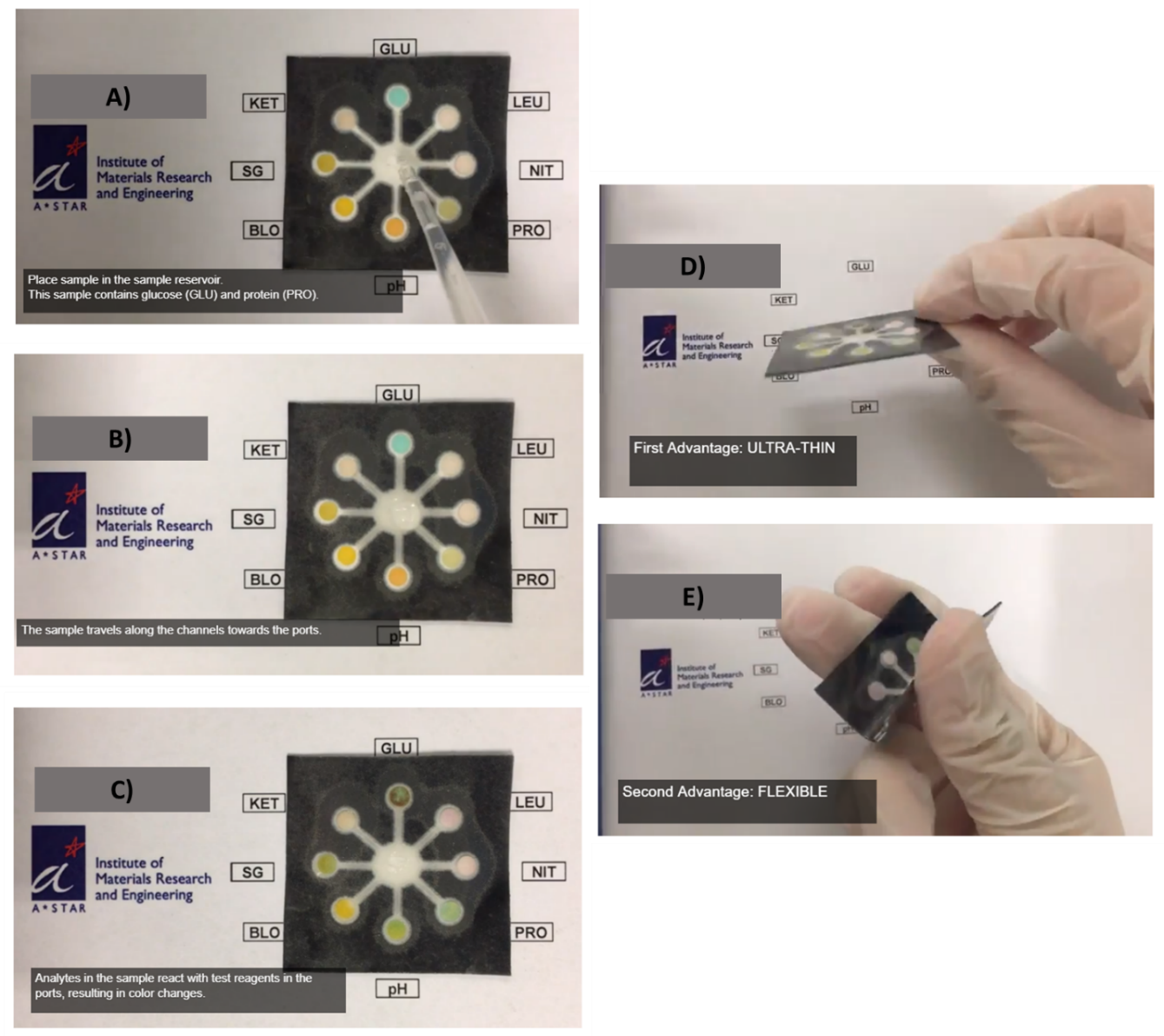


**Figure S2**. Analyte flowing through the multi-channeled paper fluidic device. The analyte after being dispensed (A) flows through the paper channels and react with the test reagents in the ports (B) resulting in color change (C). The paper based devices are ultra-thin (D) and are highly flexible (E) that they are often the preferred candidates in point-of-care applications.
